# Acute and Sub-Acute Toxicity Studies of *Plumeria alba* Linn. (Apocynaceae) Hydroalcoholic Extract in Rat

**Published:** 2013-12

**Authors:** K. Z. Tessou, P. Lawson-Evi, K. Metowogo, A. Diallo, K. Eklu-Gadegkeku, K. Aklikokou, M. Gbeassor

**Affiliations:** Department of Animal Physiology, Faculty of Sciences, University of Lome, Togo

**Keywords:** Acute toxicity, *Plumeria alba*, Sprague Dawley, subacute toxicity

## Abstract

*Plumeria alba* Linn (*Apocynaceae*) is used in Togolese traditional medicine to treat diabetes mellitus and wounds. The present investigation was carried out to evaluate the toxicity of hydroalcoholic extract of *Plumeria alba roots* in Sprague Dawley rats. The acute toxicity test was conducted by administering orally dose of 5 g/Kg. General behavior and mortality were examined for up to 14 days. The sub-acute toxicity test was performed by daily gavage at 250, 500 and 1000 mg/Kg for 28 days. Body weight and blood glucose were measured weekly. Hematological and biochemical parameters, relative organ weight were determined at the end of the 28 days administration. In acute study, no adverse effect of the extract was observed at 5.0 g/Kg. Sub-acute oral administration of the extract at the dose up to 1000 mg/Kg did not induce death or significant changes in body weight, relative weight of vital organs, hematological parameters and was not associated with liver and kidney toxicity.

## INTRODUCTION

*Plumeria alba* Linn. (*Apocynaceae*) commonly called White Fangipania, is a small latex bearing tree or shrub, native of South America. The plant is 4.5 m high, cultivated occasionally in gardens for its ornamental and fragrant flowers. In Togo and neighboring countries, the plant is also found in cemeteries in rural areas. Different part of *P. alba* are used in Indian traditional medical system for the treatment of various diseases (1, 2). The latex is applied to ulcers, herpes, scabies, wounds and the seeds possess hemostatic properties. Moreover, its bark is bruised and applied as plaster over hard tumors (2-4). Methanolic extract showed antimicrobial activity against *Bacillus anthracis* and *Pseudomonas aeruginosa* (5). Radha *et al.* (2) reported the presence of alkaloids, flavonoids, glycosides, phytosterols and terpenoids in the plant. Other studies showed that *P. alba* contains amyrinacetate, mixture of amyrins, β-sitosterol, scopotetin, the iridoids isoplumericin, plumieride, plumieride coumerate and plumieride coumerate glucoside (6, 7).

In Togo, decoction of *P. alba* root is used in folk medicine to treat hyperlipidemia, type 2 diabetes mellitus and to manage obesity and the latex to treat wound.

Although *P. alba* is worldwide used in traditional medicine, toxicological data on the plant are scarce. This study was carried out to assess the toxicity of the root hydroalcoholic extracts in Sprague Dawley rats, with the purpose that the results would provide information on the safe use of this plant.

## MATERIALS AND METHODS

### Plant material

*P. alba* roots were collected from the garden of the Teaching Hospital Sylvanus Olympio of Lomé, Togo. A specimen was identified by the Laboratory of Botany and Plant Ecology (Faculty of Science/University of Lomé) and retained in the department herbarium under number 8035. The roots were washed, dried under air-conditioning and reduced to powder with electric mill (Thomas Scientific^TM^, 3375-E20). The powder was cold extracted in ethanol 95°/water mixture (80:20) for 72 h. The crude extracts were filtered with Whatman paper (N° 1) and evaporated under vacuum at 45°C using a rotary evaporator Büchi R210. The yield of the preparation was 11.34 %.

### Animals

Male and female Sprague Dawley rats weighing 150–200 g were used in this study. The animals were housed in colony cages (8 rats per cage), under standard laboratory conditions (24°C, 30–70 % humidity, 12 h light/dark cycle) and had free access to standard commercial diet and tap water. All animal experiments were conducted under strict institutional ethical guidelines.

### Acute oral toxicity assay in Sprague Dawley (OECD 423)

Acute oral toxicity test was performed as per OECD 423 guidelines. Two groups of animals were constituted. Each group contains 8 rats (four males and four females). The first group received per os distilled water and the second group received orally 5000 mg/Kg body weight of extracts. The animals were observed for mortality, signs of gross toxicity and behavioral changes one hour post dosing and at least once daily for 14 days. Body weights were recorded before dosing and after the observation period.

### Sub-acute toxicity in Sprague Dawley (OECD 407)

Four groups of 8 animals (four males and four females) were used. Rats were treated daily for 28 consecutive days. The test groups received orally by gavage, the extracts dissolved in distilled water at the doses of 250, 500 and 1000 mg/Kg. Distilled water was given to the control animals. The animals were observed for signs of toxicity and mortality throughout the experimental period.

At the end of the 28 day experiment, the animals were fasted for 12 h. Blood was collected under light ether anesthesia from the retro orbital sinus into tubes containing EDTA for hematological examination and into anticoagulant-free tubes. The anticoagulant-free tubes were centrifuged at 3000×g for 10 min to obtain serum for biochemical analysis. The animals were then sacrificed by cervical dislocation under anesthesia. Kidneys, livers and hearts were excised and weighed.

### Blood analysis

Blood glucose was determined in all groups using the One Touch Ultra glucometer on days 0, 7, 14, 21 and 28 on caudal vein blood samples obtained from fasted rats. White blood cell (WBC), red blood cell (RBC), leukocyte and platelet counts, hematocrit, hemoglobin, mean corpuscular volume (MCV), mean corpuscular hemoglobin (MCH) and mean corpuscular hemoglobin concentration (MCHC), were determined using an automatic analyser (System H1, Bayer Diagnostics). Serum was analyzed for cholesterol, creatinine, triglycerides and transaminases activities, using commercial diagnostic kits (Labkit). Optical densities were measured using a spectrophotometer (UV spectrophotometer Hitachi).

### Statistical analysis

Data are presented as mean ± S.E.M. One-way ANOVA with Dunett’s Multiple Comparison post-test was performed to assess differences between groups (Graph PadPrism 5, San Diego, CA). Values of *P<0.05* were considered statistically significant.

## RESULTS

### Acute oral toxicity of *P. alba* root hydroalcoholic extract

No signs of toxicity (behavioral changes or mortality) were observed after single oral administration of hydroalcoholic extract (5000 mg/Kg b.w.) in rats during two weeks of observation. Therefore the LD_50_ of oral administration of *P. alba* extract is higher than 5000 mg/Kg in Sprague Dawley rats.

### Sub-acute oral toxicity of *P. alba* root hydroalcoholic extract

**Figure 1:**
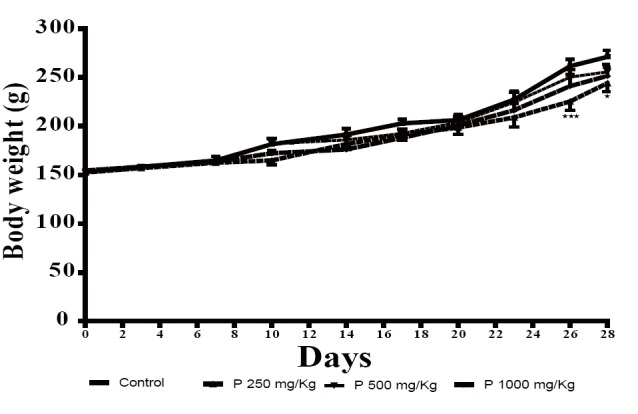
Effect of sub-acute oral administration of *P. alba* hydroalcoholic extract on body weight of rats. Each point represents mean ± S.E.M. *p<0.05; ***p<0.001: Vs control. C: control; P250 mg/Kg, P 500 mg/Kg and P1000 mg/Kg: received respectively *P. alba* extract at 250, 500 and 1000 mg/Kg.

Daily oral administration of *P. alba* root hydroalcoholic extract at all tested doses (250, 500 and 1000 mg/Kg b.w.) for 28 days, did not induce any obvious symptoms of toxicity and mortality in rats of both sexes. Figure 1 shows a significant reduction in weight gain in animals treated with 250 mg/Kg compared to normal controls.

*P. alba* extract at all the doses tested (250, 500 and 1000 mg/Kg b.w.) reduced basal blood glucose level. However, only the low dose (250 mg/Kg b.w.) induced a significant reduction over the duration of the experiment (Table 1).

**Table 1:** Effect of hydroalcoholic extract of *Plumeria alba* on blood glucose level after 28 days administration

Groups	Blood glucose level (mg/dL)				
	Day 0	Day 7	Day 14	Day 21	Day 28

Control	99.75 ± 1.81	98.62 ± 3.12	97.12 ± 1.40	96.12 ± 2.68	92.37 ± 2.78
P 250 mg/Kg	99.75 ± 1.81	88.25 ± 1.91^a^	84.25 ± 2.38^b^	83.75 ± 3.35^b^	75.62 ±1.42^c^
P 500 mg/Kg	99.75 ± 1.81	90.50 ± 1.61	86.75 ± 3.47^a^	88.37 ± 2.35	87.75 ± 3.54
P 1000 mg/Kg	99.75 ± 1.81	86.12 ± 3.71^a^	90.12 ± 2.29	87.62 ± 2.98	84.75 ± 2.72

Each data represents mean ± SEM of 8 rats. Blood glucose levels were measured before the administration of the extract on day 0 and on days 7, 14, 21 and 28.

a *p*<0.05;

b *p*<0.01;

c *p*<0.001 *vs Control*.

Relative organ weights after 28 day treatment were shown in Table 2. There was no significant change in the organ weights of the treated groups compared to the control.

**Table 2:** Effect of sub-acute oral administration of hydroalcoholic extract of Plumeria alba on organ weights of rats

Organs	Relative organ weight (g per 100 g body weight)			
	Control	P 250 mg/Kg	P 500 mg/Kg	P 1000 mg/Kg

Liver	4.9 ± 0.19	3.4 ± 0.12	3.7 ± 0.18	3.5 ± 0.13
Heart	0.40 ± 0.13	0.41 ± 0.11	0.39 ± 0.024	0.43 ± 0.07
Kidney	0.56 ± 0.05	0.54 ± 0.07	0.55 ± 0.09	0.60 ± 0.11

Data are expressed as mean ± S.E.M. (8 rats). Hydroalcoholic extract of *Plumeria alba* was administrateddaily by gavage for 28 days. There was no significant difference.

The effect of sub-acute administration of *P. alba* root hydroalcoholic extract on hematological parameters was presented in Table 3. No difference was observed between the treated and control groups.

**Table 3:** Effect of sub-acute oral administration of hydroalcoholic extract of *Plumeria alba* on hematological parameters in rats

Parameters	Control	P 250 mg/Kg	P 500 mg/Kg	P 1000 mg/Kg

WBC (10^3^/μL)	5.8 ± 0.72	4.4 ± 1.8	4.9 ± 1.5	4.7 ± 0.33
RBC (10^6^/μL)	6.9 ± 0.42	7.1 ± 0.62	7.3 ± 0.55	7.7 ± 0.33
Hb (g/dL)	12.9 ± 0.45	12.5 ± 0.22	12.2 ± 0.32	12.2 ± 0.31
Ht (%)	40.7 ± 0.03	40.2 ± 2.33	40.5 ± 2.27	40.1 ± 2.23
MCV (fl)	51.5 ± 0.99	49.1 ± 0.58	49.1 ± 0.58	49.6 ± 0.61
MCH (pg)	17.2 ± 0.17	16.12 ± 0.11	16.1 ± 0.21	16.3 ± 0.27
MCHC (%)	34.5 ± 0.17	33.22 ± 1.34	32.9 ± 1.39	33.40 ± 0.98
Plaquettes (10^3^/μL)	763 ± 73	633 ± 65	665 ± 47	642 ± 68

Data are expressed as mean ± S.E.M. of 8 rats.No significant difference in hematological parameter was observed between tested and control groups.

Biochemical parameter profiles of the treated and control groups are shown in Table 4.

**Table 4:** Effect of sub-acute oral administration of hydroalcoholic extract of *Plumeria alba* on biochemical parameters of rats

Parameters	Control	P 250 mg/Kg	P 500 mg/Kg	P 1000 mg/Kg

ASAT (UI/L)	133 ± 20	122 ± 23	128 ± 22	118 ± 25
ALAT (UI/L)	37 ± 3	25 ± 7	29 ± 9	30 ± 12
GGT (UI/L)	2.66 ± 0.4	3.59 ± 0.5	3.34 ± 0.8	3.38 ± 0.9
CK (UI/L)	369 ± 130	558 ± 49	534 ± 78	433 ± 139
Urea (mmol/L)	4.03 ± 0.72	4.07 ± 0.87	4.20 ± 0.64	4.15 ± 0.25
Creatinine (μmol/L)	43.4 ± 4.5	33.4 ± 4.0	35.4 ± 5.0	41.7 ± 3.6
Chol (mg/dL)	77.3 ± 4.40	70.8 ± 3.1	70.2 ± 3.1	70.6 ± 4.3
HDL-Chol (mg/dL)	30.32 ± 1.23	57.33 ± 4.6^b^	48.27 ± 3.26^a^	43.52 ± 2.25^a^
TG (mg/dL)	32.66 ± 1.77	18.11 ± 3.57^a^	29.11 ± 5.2	29.73 ± 5.23

Data are expressed as mean ± S.E.M. of 8 rats.

a *p<0.5*;

b *p<0.01*;

The 28 day oral administration of thehydroalcoholic extract did not cause significant changes in serum creatinine, cholesterol, and transaminase activities (ALT/AST). In the group treated with 250 mg/Kg of extract, triglycerides levels were significantly lower (*p*<0.05) compared to the control while in the high dose (500, 1000 mg/Kg b.w.) the reduction of triglycerides level is not significant. HDL-Cholesterol was higher (*p*<0.01) in treated groups compared to control.

## DISCUSSION

Traditional remedies containing essentially medicinal and aromatic plants are used by the majority of the population in Subsaharan Africa. Despite this widespread use, scientific studies designed to validate their efficacy and evaluate their safety are few.

The present investigation was carried out to estimate safety limits of oral administration of the hydroalcoholic extract of *P. alba*.

In the oral acute toxicity study, rat administered 5.0 g/Kg did not exhibit any sign of adverse effect. The LD50 for the oral administration of hydroalcoholic extract of *P. alba* roots was estimated to be >5000 mg/Kg. In this case, according to OECD directive, the oral administration of hydroalcoholic extract of *P. alba* roots could be considered practically non-toxic or at worst slightly toxic (8).

In the sub-acute toxicity study, *P. alba* hydroalcoholic extract was administered at the doses of 250, 500 and 1000 mg/Kg b.w. The results showed that there were no significant changes in animal behavior, relative weight of liver, kidney and heart in treated animals compared to control animals. The low dose (250 mg/Kg) induced a significant reduction in weight compared to the control. However, this reduction was not dose dependent and therefore was not considered as a toxic effect.

Our result showed that the effect of *P. alba* extract on glucose level faded at higher doses. This finding was also observed in our previous study evaluating the effect of *P. alba* extract on metabolic syndrome in rat (unpublished data). This phenomenon could be due to the presence of substances in the extract inducing or enhancing their own metabolism and consequently the elimination of the extract (9).

Blood triglycerides level was also significantly reduced only by 250 mg/Kg. Doses 500 and 1000 mg/Kg reduced marginally triglycerides level. All the doses (250, 500 and 1000 mg/Kg) increased significantly HDL-cholesterol. In the light of this result, 250 mg/Kg seems to be a pharmacological dose and results obtained after administration of 500 and 1000 mg/Kg may suggest saturation effect which could show toxic impact in longer exposure (subchronic toxicity test). It is reported that, HDL is one of the major groups of lipoprotein which is positively associated with a decreased risk of coronary heart disease (10). Therefore effect of *P. alba* extract at 250 mg/Kg on lipid parameters could be considered as non toxic.

The extract did not alter serum transaminase activities (ALT/AST) and creatinine suggesting that sub-acute administration of *P. alba* extract was not associated with liver and kidney toxicity.

No significant alterations of the hematological parameters of both male and female treated rats can be attributed to the plant extract.

## CONCLUSION

In conclusion, the 28-day sub-acute toxicity test revealed no adverse effects attributable to oral administration of *P. alba* hydoalcoholic extract at 250 g/kg. Further studies are required to elucidate the fading of the effect of *P. alba* extract on blood glucose and triglycerides at 500 and 1000 g/Kg in Sprague Dawley rats. This study provides data for further investigations on detailed toxic effects of this plant and its safe use in human.

## SOURCE OF SUPPORT

Fellowship from French Cooperation Service (SCAC).
